# Opening of the Woman’s Medical College, New York

**Published:** 1868-12

**Authors:** 


### Opening of the Woman's Medical College, New York.

The Woman's Medical College of the New York Infirmary, 126 Second Avenue, opened its first session December 2d.

The introductory address was delivered by Dr. Elizabeth Blackwell. She spoke of the slow and silent growth of all great movements at their beginning; alluded to the first projection of a medical institution for women, in a private parlor, 1853; to the actual establishment of a small hospital in Bleecker street, in 1857; and rejoiced at length to see the opening of the present long-desired college. To some the interval of fifteen years might seem needlessly long; and doubtless the promoters might have done as others did, and opened long since a college at which women might have received learned-looking parchments entitling them to the degree of M. D. But a *poor* college was no desideratum to them; and it had been impossible before the present time to found a medical school wherein women should receive a thoroughly good education; which should issue diplomas commanding the respect of the whole profession. The doctor spoke strongly of the responsibility incurred by sending forth unqualified women as physicians, and argued that a long and thorough course of study was the only safeguard against the temptation of running unprepared into practice, to which women were even more exposed than men. After commenting on the extreme difficulty of raising funds for the establishment of a principle not yet popular, and on the obstacles thrown in the way of obtaining able professional aid by prejudices lately general, she expressed her satisfaction that at length a solid, though small, pecuniary basis had been secured; and referred to the list of the Faculty in proof that prejudice was no longer able to deprive women of the best medical instruction. She congratulated New York on being the first to establish such a college for women; quoted the remarks of an eminent Boston physician, who regretted that the initiation had not been taken by his own city; and mentioned the satisfaction expressed by the really qualified medical women, in all parts of the country, that students should now be relieved from many of the difficulties with which they themselves had contended.—*Medical Record.*

